# Anticoagulation Monitoring During ECMO Support: Monitor or Flip a Coin?

**DOI:** 10.1002/clc.70061

**Published:** 2024-12-18

**Authors:** Sasa Rajsic, Benedikt Treml

**Affiliations:** ^1^ Department of Anaesthesiology and Intensive Care Medicine Medical University Innsbruck Innsbruck Austria

## Abstract

Should we rely on anticoagulation monitoring in ECMO patients or simply flip a coin? The increasing use of anti‐factor Xa activity to monitor the effect of UFH appears appropriate, given its moderate correlation with the UFH infusion rates, and it may play a role in preventing thromboembolic events. However, to avoid bleeding complications, more sophisticated tools, and careful clinical decision‐making remain essential.

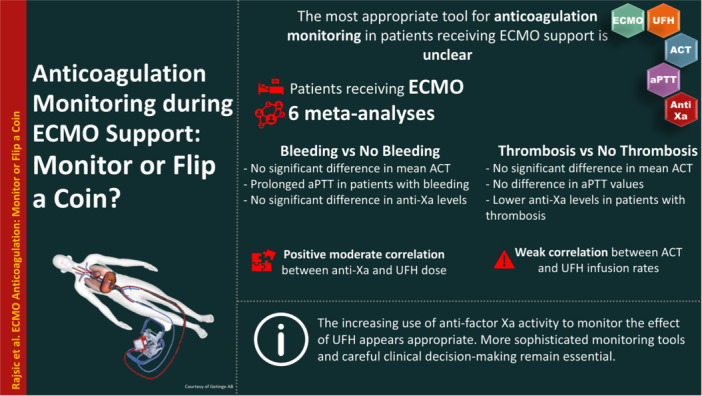

Extracorporeal membrane oxygenation (ECMO) is an advanced life support modality for patients with refractory cardiac or respiratory failure. Its initiation is associated with complex inflammatory and coagulatory processes, requiring systemic anticoagulation to maintain circuit patency and reduce the risk of thromboembolic events or circuit component failure. However, the use of therapeutic anticoagulation in critically ill patients carries an additional risk of potentially life‐threatening hemorrhage. Therefore, monitoring of anticoagulation is crucial to ensure the balance of anticoagulatory and procoagulant factors [1].

The Extracorporeal Life Support Organization (ELSO) recommends the use of activated clotting time (ACT), activated partial thromboplastin time (aPTT), viscoelastic tests, or anti‐factor Xa assays [2, 3]. The International Society on Thrombosis and Hemostasis (ISTH) recommends anti‐factor Xa‐guided unfractionated heparin (UFH) monitoring, ACT, and aPTT remain as an alternative [4]. However, the evidence on anticoagulation monitoring in patients receiving ECMO support is weak, and there is an ongoing need to develop more appropriate monitoring tools.

An international survey from 2011 showed that ACT was the preferred method for anticoagulation monitoring, as reported by 97% of centers (113 out of 116) [5]. The routine or occasional use of anti‐factor Xa was reported by 65% of centers. Seven years later, a follow‐up survey revealed a shift toward the use of aPTT [6]. Protti et al. reported on 273 centers from 50 countries, showing that aPTT was used in the majority of centers (42%, 114 centers), followed by ACT (30%, 82 centers) and anti‐factor Xa (23%, 62 centers). In subsequent years, increasing evidence on patients receiving ECMO support has emerged, making it possible to systematize data from studies. Rajsic et al. investigated the role of time‐guided anticoagulation monitoring tools, including anti‐factor Xa, in ECMO patients and highlighted the strengths and limitations of widely used and available methods [7, 8, 9, 10, 11, 12]. In this editorial, we summarize the most recent evidence.

## 1 ACT and aPTT

ACT assesses contact activation and intrinsic anticoagulation by heparin or direct thrombin inhibitors. Despite its low cost, point‐of‐care availability, and short turnaround time, ACT is now considered a rather historical test for anticoagulation monitoring during ECMO. The most recent systematic review and meta‐analysis reaffirmed its questionable role, showing no significant difference in mean ACT values between patients with and without hemorrhagic or thromboembolic events [10]. Moreover, a meta‐analysis of correlation coefficients, which included 19 articles with 12 625 samples, demonstrated a weak correlation between ACT and UFH infusion rate. None of the studies reported a strong correlation. The authors concluded that although ACT is a widely used tool for UFH monitoring, the evidence regarding its association with hemostatic complications remains controversial and limited [10].

Regarding aPTT, the authors confirmed that aPTT‐guided systemic anticoagulation remains the standard monitoring tool for ECMO patients [9, 11]. The availability of point‐of‐care assays has significantly reduced turnaround time from nearly 1 h to just 5 min [13]. However, the authors did not find strong evidence supporting any specific aPTT threshold for anticoagulation during ECMO. Available studies reported prolonged aPTT in patients experiencing hemorrhage, including longer duration of ECMO support. Although the meta‐analysis included data from 3249 ECMO patients, its findings were limited by the retrospective nature of the included studies and high heterogeneity [11]. In cases of thrombosis in patients receiving V‐A ECMO support, the authors did not find any difference in reported aPTT values between those with and without thrombotic events. Furthermore, within the qualitative analysis, none of the studies found an association between aPTT and thrombosis. This analysis was constrained by the small number of studies (six) involving 1728 patients, as well as the retrospective, single‐center nature of the included works [9].

Systematized evidence on the correlation between aPTT and UFH infusion rates in ECMO patients is still missing, representing a significant research gap for future studies.

## 2 Anti‐Factor Xa

Anti‐factor Xa assays, which measure the ability of heparin‐bound antithrombin to inhibit factor Xa, are gaining popularity as an important component of UFH monitoring in patients receiving ECMO support [1]. A meta‐analysis of anti‐Xa‐guided anticoagulation monitoring, encompassing 26 articles with a total of 2293 patients, did not find a significant difference in anti‐Xa levels between patients with and without hemorrhagic events [7]. However, the authors found a moderate correlation between UFH dose and anti‐Xa levels, supporting its utility in anticoagulation monitoring [7]. Additionally, in cases of thromboembolic events (16 studies, 1968 patients), anti‐Xa levels were significantly lower in patients experiencing thrombosis [8]. Both meta‐analyses were limited by the predominantly retrospective nature of the included studies and the relatively small number of studies examining thromboembolic events. Despite its longer turnaround time compared to time‐guided methods, anti‐Xa demonstrates potential, and further research is warranted.

Although significant advancements have been made in critical care, time‐guided anticoagulation monitoring tools remain widely used in patients receiving ECMO support. Considering the contemplative limitations of these methods, combining multiple tools may be the most effective approach to minimizing the risk of adverse events.

Given the above, should we rely on monitoring or simply flip a coin? The increasing use of anti‐factor Xa activity to monitor the effect of UFH appears appropriate, given its moderate correlation with the UFH infusion rates, and it may play a role in preventing thromboembolic events. However, to avoid bleeding complications, more sophisticated tools, and careful clinical decision‐making remain essential. The role of viscoelastic methods remains to be clarified. Based on the current evidence, further retrospective and prospective studies, including randomized trials, are crucial to identifying the optimal monitoring strategy.

## Ethics Statement

The authors have nothing to report.

## Consent

The authors have nothing to report.

## Conflicts of Interest

The authors declare no conflicts of interest.

## Data Availability

Data sharing is not applicable to this article as no data sets were generated or analyzed during the current study.
